# Coexistence between renal cell cancer and Hodgkin's lymphoma: A rare coincidence

**DOI:** 10.1186/1471-2490-6-10

**Published:** 2006-03-20

**Authors:** Victor H Jimenez I

**Affiliations:** 1 Department of Hematology and Oncology, Instituto Nacional de Ciencias Médicas y Nutrición Salvador Zubirán, Vasco de Quiroga 15, Colonia seccion XVI, Tlalpan, DF, CP 14,000, México

## Abstract

**Background:**

Renal cell carcinoma is the most common kidney tumor in adults and accounts for approximately 3% of adult malignancies. An increased incidence of second malignancies has been well documented in a number of different disorders, such as head and neck tumors, and hairy cell leukemia. In addition, treatment associated second malignancies (usually leukemias and lymphomas but also solid tumors) have been described in long term survivors of Hodgkin's lymphoma (HL), Non Hodgkin's lymphoma and in various pediatric tumors.

**Case presentation:**

We present the case of a 66 year-old woman with abdominal pain and dyspnea. We performed a thorax CT scan that showed lymph nodes enlargement and subsequently by presence of abdominal pain was performed an abdominal and pelvis CT scan that showed a right kidney tumor of 4 × 5 cms besides of abdominal lymph nodes enlargement. A radical right nephrectomy was designed and Hodgkin's lymphoma was diagnosed in the abdominal lymph nodes while renal cell tumor exhibited a renal cell cancer. Patient received EVA protocol achieving complete response.

**Conclusion:**

We described the first case reported in the medical literature of the coexistence between Hodgkin's lymphoma and renal cell cancer. Previous reports have shown the relationship of lymphoid neoplasms with solid tumors, but they have usually described secondary forms of cancer related to chemotherapy.

## Background

Renal cell carcinoma is the most common kidney tumor in adults and accounts for approximately 3% of adult malignancies. The incidence is higher in male and appears to be increased with cigarrete use and with obesity.[1,2] There are both familial and sporadic forms of renal cell carcinoma. Patients with von Hippel-Lindau (VHL) disease have a predisposition to develop bilateral renal tumors among other malignancies, VHL gene has been identified on the short arm of chromosome 3 (3p). [3] An increased incidence of second malignancies has been well documented in a number of different disorders, such as head and neck tumors, [4,5] and hairy cell leukemia. [6] In addition, treatment associated second malignancies (usually leukemias and lymphomas but also solid tumors) have been described in long term survivors of Hodgkin's lymphoma (HL) [7,8], Non Hodgkin's lymphoma (NHL) [9-11] and in various pediatric tumors. [12,13] There have been sporadic case reports of hairy cell leukemia [14], chronic myeloid leukemia [15], multiple myeloma [16,17], NHL [18], HL [19] and testicular carcinoma.[20] Nishikubo et al, described a serie of eight patients with renal cell cancer and lymphoid malignancies. One of these patients included the association between renal cell cancer and HL but the presentation did not occur at the same time 21. We describe the presentation at the same time of both entities.

## Case presentation

**Figure 1:**
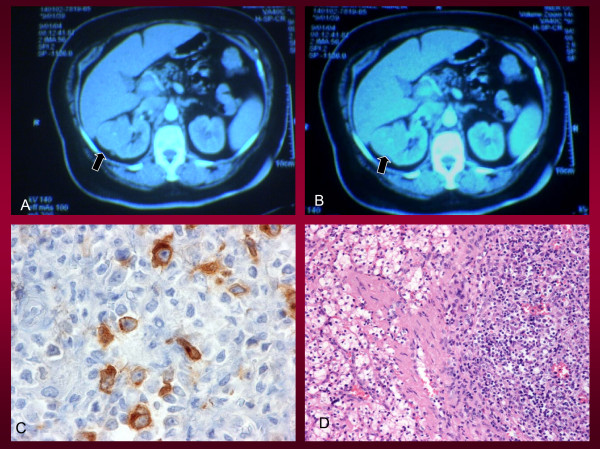
Panel A and B shows a tumor involving the right kidney, measuring 4 × 5 cms. Panel C shows the CD 30 immunostain being positive in the neoplasms cells and Panel D shows a transitional area between renal cell cancer and Hodgkin's lymphoma.

We present the case of a 66 year-old woman with history of intense smoking (pack years of 50), diabetes mellitus detected a prior year and COPD (Chronic obstructive pulmonary disease) diagnosed 8 prior months. She came at the emergency department to receive medical attention by presenting fever, loss of weight and dyspnea. During the initial evaluation was performed a thorax CT scan that showed lymph nodes enlargement and subsequently by presence of abdominal pain was performed an abdominal and pelvis CT scan that showed a right kidney tumor of 4 × 5 cms besides of abdominal lymph nodes enlargement. A radical right nephrectomy was designed, prior to surgery we performed an spirometer study that revealed a moderate obstruction pattern. The surgical procedure included lymph nodes remotion. Histopathology study revealed Hodgkin's lymphoma involving right kidney, abdominal lymph nodes, ureter and liver. Immunostaining showed positiveness for CD15, CD30 and LMP-1 while CD3, EMA and ALK were negative. Histological study also reported a clear renal cell cancer, degree II, involving only the right kidney without extension to any other structure. Surgical resection was complete and margins were negative for cancer, diffuse glomerulosclerosis also was found. After surgical procedure we did a bilateral bone marrow biopsy that was negative for lymphoma. Staging studies showed an IV b stage and 4 unfavorable factors (stage IV, hemoglobin 7,2 g/dl, albumin 3.2 g/dl and lymphocytes <0.6) at diagnosis. We performed an echocardiography study that revealed light enlargement of right cavities, diastolic dysfunction and moderate pulmonary hypertension. The patient received chemotherapy with a protocol called EVA (etoposide, vinblastine and doxorubicin). After third cycle we documented complete response, fact that was obtained after completed the chemotherapy scheme. At present she continues asymptomatic without evidence of disease activity. We present a figure 1 that shows in the panel A and B a tumor involving the right kidney, measuring 4 × 5 cms while panel C shows the CD 30 immunostain being positive in the neoplasms cells and panel D shows a transitional area between renal cell cancer and Hodgkin's lymphoma.

## Conclusion

We described the first case reported in the medical literature of the coexistence between Hodgkin's lymphoma and renal cell cancer. Previous reports have shown the relationship of lymphoid neoplasms with solid tumors, but they have usually described secondary forms of cancer related to chemotherapy. Nishikubo et al, reported a case of a 63 year-old man that developed renal cell cancer 8 years after treatment for Hodgkin's lymphoma. [21] Due to the presence of pulmonary hypertension and lung injury related to COPD we decided to use the EVA protocol to treat this patient. Canellos et al, described the employment of the EVA protocol to treat HL patients as a first line therapy including advances stages, 51 patients, even 12 with bulky disease received this protocol, 10 of which received concomitant radiotherapy. [22] EVA protocol achieved complete response in 94% and free progression survival of 57% at 111 months since diagnosis. Our patient is free disease at 2 months since the last course of chemotherapy. She presented 4 unfavorable factors, fact that predicts a possible recurrence. Multiple cancers developing after chemotherapy and radiotherapy have been described but we presented a peculiar case of simultaneous presentation of a solid tumor an HL. [23,24] It is worthwhile to look for second malignancies and get additional biopsies in case of unclear finding. This coincidence appears to be rare. We diagnosed annually 10 cases of renal cell carcinoma and 20 cases a year of Hodgkin's lymphoma.

## Competing interests

The author(s) declare that they have no competing interests.

## Authors' contributions

VJ participated designing the study, collecting data, reviewing and writing the article.

## Pre-publication history

The pre-publication history for this paper can be accessed here:
